# Inverted Papilloma From Nasal Septum: A Rare Case Presentation

**DOI:** 10.7759/cureus.48486

**Published:** 2023-11-08

**Authors:** Jasleen Kakkad, Prasad Deshmukh, Sagar Gaurkar

**Affiliations:** 1 Otolaryngology, Head and Neck Surgery, Jawaharlal Nehru Medical College, Datta Meghe Institute of Higher Education and Research, Wardha, IND

**Keywords:** septal perforation, nuclear atypia, nasal septum, inverted sinonasal papilloma, nasal mass

## Abstract

Inverted sinonasal papillomas, also referred to as Schneiderian papillomas, are benign tumors originating from the Schneiderian membrane that lines the nasal cavity and paranasal sinuses. They frequently display an endophytic growth pattern, in which the stroma beneath is invaded by epithelial cells. The exact cause of inverted sinonasal papillomas is unknown, but several theories have been offered. The most widely accepted theory states that these tumours arise from the metaplasia of the respiratory epithelium into a stratified squamous epithelium. This metaplastic process is thought to be brought on by irritant exposure, chronic inflammation, or viral infections like the human papillomavirus (HPV). While inverted sinonasal papillomas commonly arise from the paranasal sinuses and lateral walls of the nasal cavity, their occurrence from the nasal septum is relatively rare. Additionally, although inverted sinonasal papillomas are typically benign, they can exhibit locally aggressive behaviour and damage nearby structures. The histopathological examination revealed nuclear atypia, which raises questions about the potential for malignant transformation. We describe a rare case of an inverted sinonasal papilloma that developed from the nasal septum. The tumour spread into the septum's anterior cartilaginous region, causing the cartilage to deteriorate and develop mucosal defects. The rarity of an inverted sinonasal papilloma arising from the nasal septum along with its impact on cartilaginous septum is discussed. Careful monitoring and prolonged follow-up are therefore necessary to spot any signs of recurrence or malignant changes.

## Introduction

An inverted papilloma is a benign epithelial growth that invades the stroma of the paranasal sinus and nasal cavity. It stands out due to its invasiveness, tendency to recur, and relationship to cancer. The histopathology was elaborately described by Reingertz as an inverted growth in the underlying connective tissue stroma in 1935 [1]. Inverted papilloma originating from the nasal septum is indeed a rare occurrence, and its presentation represents a unique clinical puzzle. By unraveling the intricacies of this case, we aim to contribute to the growing body of knowledge surrounding this rare nasal pathology, providing valuable insights for healthcare providers and researchers alike.

## Case presentation

An 85-year-old man visited the ENT outpatient department with complaints of chronic mucoid nasal discharge for eight months and nasal obstruction that mainly had affected his right nostril for the previous three months. The symptoms had worsened over the past 10 days, prompting him to seek medical attention. A diagnostic nasal endoscopy revealed a greyish mass in the right nasal cavity that extended from the vestibule to the region between the lateral wall and septum and was covered in slough and mucopurulent discharge. The patient was started on antibiotics and a biopsy was planned. After performing a biopsy, the specimen's histopathological analysis supported the diagnosis of inverted sinonasal papilloma with nuclear atypia (Grade II).

**Figure 1:**
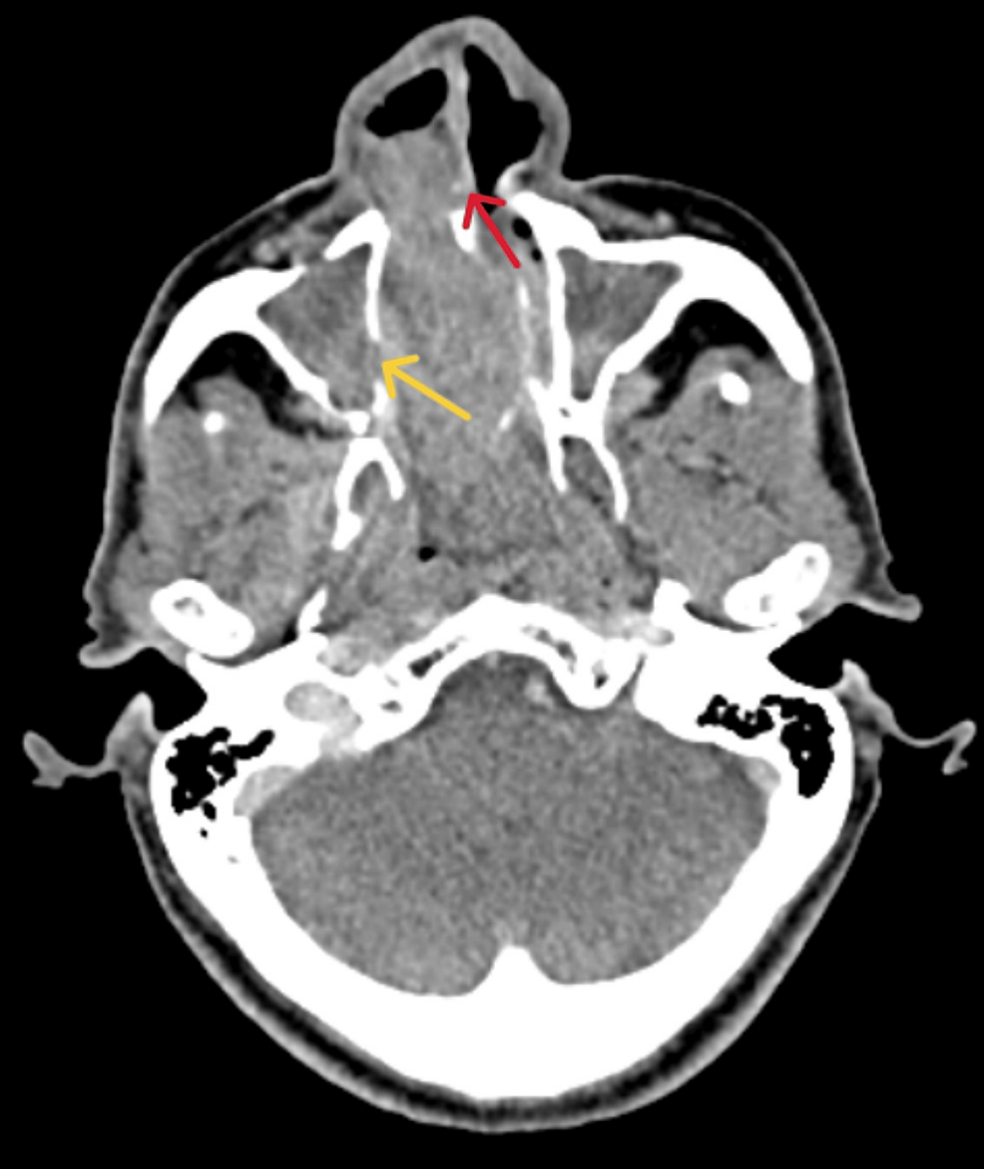
CT paranasal sinus (axial view) CT- Computed tomography A large soft tissue density mass lesion is seen in both nasal cavities, mainly in the right nasal cavity, resulting in near complete opacification with destruction of the nasal septum (red arrow) and widening of both maxillary ostia with destruction of right side maxillary wall (yellow arrow) and lamina papyracea. Complete opacification of all sinuses except left frontal sinus findings are suggestive of sinonasal mass as inverted papilloma.

Computed tomography (CT) of the paranasal sinuses (Figure 1) demonstrated near complete opacification of the nasal cavities, with the right nasal cavity being predominantly affected. The computed tomography (CT) scan also revealed destruction of the nasal septum, widening of the maxillary ostia, and near complete opacification of all sinuses except the left frontal sinus. No evident erosion of the sinus walls or intracranial extension was observed.

**Figure 2:**
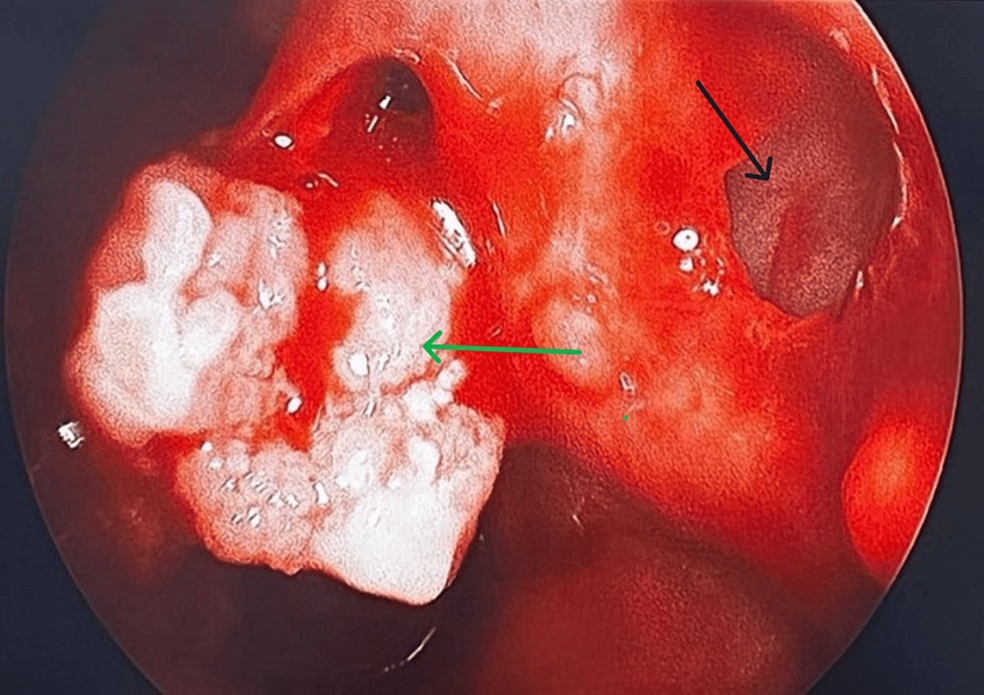
Intraoperative image of the right nasal cavity The image shows a mass (green arrow) arising from the nasal septum and perforation in the cartilaginous part of the septum (black arrow).

Given the extent of the tumour and the involvement of the anterior cartilaginous part of the septum, the patient underwent endoscopic excision of the tumour under general anaesthesia. Intraoperatively, a grayish mass covered with slough and mucopurulent discharge was visualized in the right nasal cavity, arising from the septum with septal perforation at certain areas with the destruction of the cartilaginous part of the septum (Figure 2).

**Figure 3:**
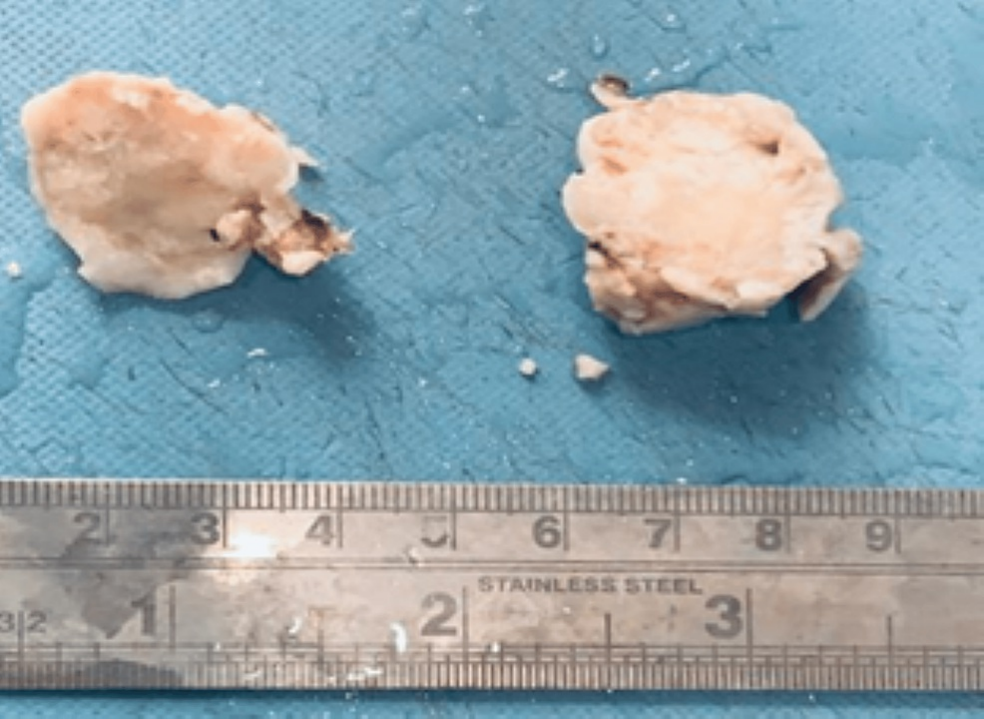
Resected nasal masses The image shows resected nasal masses, greyish white, solid, papillary growth pattern measuring 3 x 2.5 cm.

The tumor was found to reach up to the nasopharynx, and additional resection was performed. The mass was removed using a debrider, and frozen sections were sent for analysis. The specimen of the excised tumour (Figure 3) was sent for additional histopathological analysis.

**Figure 4:**
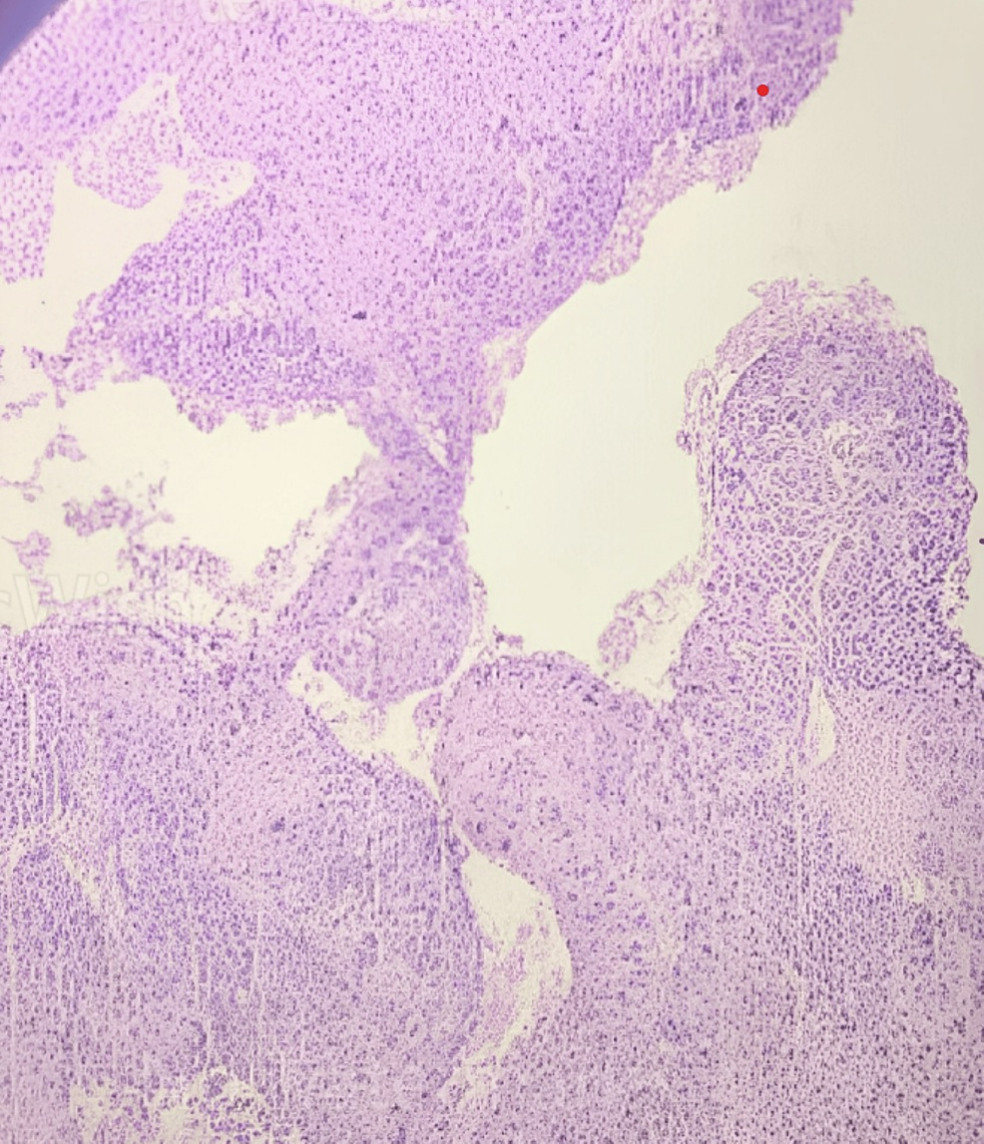
Photomicrograph of histopathological specimen at low power (4X) view The image shows nuclear atypia.

**Figure 5:**
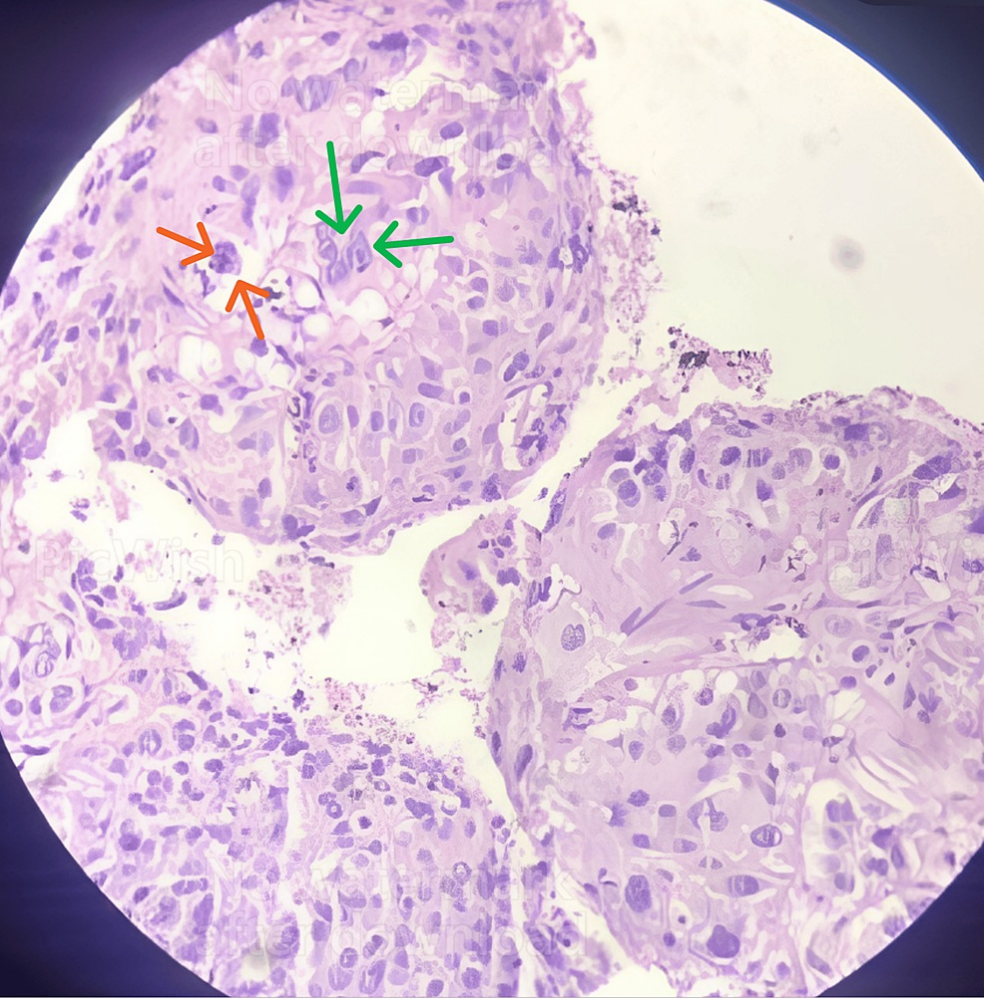
Photomicrograph of histopathological specimen in high power view (40X) The image shows nuclear atypia (green arrows) with mitosis (orange arrows).

The histopathology report showed (Figures 4-5) that columnar epithelial cells had grown downward and formed true papillae with a fibrovascular core. Some areas showed pleomorphism, hyperchromasia, atypical mitosis, loss of polarity, foci of necrosis, and foci of hemorrhage. The stroma was unremarkable. Histopathological examination done in low (Figure 4) and high (Figure 5) power field shows nuclear atypia and mitosis. Immunohistochemistry was done which showed diffuse positivity for cytokeratin.

Postoperatively, the patient had a fast recovery and followed up regularly to monitor the course with no evidence of reoccurrence.

## Discussion

The first description of inverted papilloma (IP) was written in 1854. It is a type of tumour that mostly affects the nasal cavities and paranasal sinuses. Inverted papilloma is distinguished from other sinonasal tumours by its proclivity for local aggression, high recurrence rates (both early and late), and potential association with carcinoma either at initial presentation or during recurrence [1]. Schneiderian papilloma is classified into three subtypes by the World Health Organisation (WHO): inverted, oncocytic, and exophytic, with inverted papilloma accounting for approximately 62% of sinonasal papillomas [2]. Inverted papilloma is found in 0.4% to 7% of sinonasal tumours [2]. The male-to-female ratio ranges between 2:1 and 5:1 [2]. The majority of inverted papilloma cases are diagnosed in adults, with the average age of diagnosis being around 55 years [1-3]. In our case, the patient started complaining at 85 years of age.

During endoscopic nasal probing, it reveals a reddish-gray lobulated tumor, firmer than an inflammatory polyp and with a distinct "raspberry" appearance. Inverted papillomas are notoriously friable and bleed when touched. A pathologic examination is required to make a diagnosis. In this type of papilloma, the term "inverted" refers to the superficial inverted papilloma epithelium invading the underlying stroma as seen under a microscope. The epithelium can be keratinized squamous, respiratory, or transitional depending on the proportions (Figure 3). The stroma beneath the hyperplastic inverted epithelium is normal, and the basal membrane is still intact. There may occasionally be exo- and/or endophytic components in the IP [1].

Several studies have suggested that inverted papillomas may be caused by a virus, and tests for the presence of human papillomavirus (HPV) have been performed using in situ hybridization and polymerase chain reaction. Human papilloma virus (HPV) 6, HPV 11, HPV 16, HPV 18, and Epstein-Barr virus have all been identified in isolated cases. The relationship between the presence of HPV and the development of inverted papillomas, on the other hand, remains unknown [4]. Overexpression of oncoproteins E6 and E7 caused by HPV integration into the cell genome can deactivate cell-cycle regulators such as p16, p21, p27, p53, cyclin D1, and the Rb protein [1]. In contrast to benign IP or healthy mucosa, p53 has been reported only present in IP linked with malignancies [5,6]. Various mechanisms are believed to be connected: either a mutation in the p53 gene or a rise in the normal p53 protein's degradation, both of which reduce p53's ability to suppress tumours.

Inverted papillomas are most commonly found along the lateral nasal wall, specifically in the middle turbinate or ethmoid recesses, and can spread into the paranasal sinuses [4]. They may begin in the paranasal sinuses, with or without nasal cavity involvement. The lateral nasal wall is the most common primary site of origin for inverted papillomas (89%), followed by the maxillary sinus (53.9%) and ethmoid sinuses (31.6%) [7,8]. The septum (9.9%), frontal sinus (6.5%), and sphenoid sinus (3.9%) are also less common sites. Symptoms of inverted papillomas include unilateral nasal blockage (58%), epistaxis (17%), nasal discharge (14%), and sinusitis (9%). Headache, facial numbness, oedema, diplopia, and anosmia are some of the other symptoms. Most cases of IPs are unilateral, although approximately 4.9% of patients may have bilateral lesions [8]. If bilaterality is present, clinical evaluation should be done to rule out the possibility of unilateral disease extension [9].

Sinonasal inflammatory polyps, verrucous carcinoma, and nonkeratinizing respiratory cancer are all included in the differential diagnosis for inverted papilloma [10]. Sinonasal inflammatory polyps and inverted papillomas can be distinguished from one another by histological analysis even though they may share some clinical characteristics. Inverted papillomas have epithelial changes that inflammatory polyps do not. Despite nonkeratinizing respiratory carcinoma can resemble inverted papillomas, the cancer's dysplastic features assist in distinguishing it from the latter. Verrucous carcinoma can be differentiated from IP by the characteristic presence of cleft-like gaps within the lesion. These gaps are lined by a thick layer of parakeratin, which extends deeply into the lesion [11].

The preoperative assessment of patients with Inverted papillomas is extremely important. Computed tomography (CT) is frequently utilised and can offer useful details regarding the nature and extent of the tumour and frequently show up as heterogeneously increasing soft-tissue densities. However, computed tomography (CT) scans might not be able to distinguish between the tumour and nearby inflammatory changes or secretions in order to pinpoint the exact location of attachment. The ability of MRI to discern between the tumour and the inflammatory tissue around it has increased, making it a more precise staging method [8]. The presence of sclerosis, calcification, lobulation, and bone erosion on a CT scan can also indicate the presence of inverted papillomas. These characteristics can help in the diagnosis of sinus involvement, along with opacification. Inverted papillomas can be distinguished from benign nasal polyps on CT scans by looking for histologic abnormalities in the bone nearby, such as periosteal thickening and osteoblastic rimming, which can also assist in pinpointing the attachment site F-18 fluorodeoxyglucose positron emission tomography (FDG-PET) has been utilised for staging and post-treatment monitoring of inverted papillomas with squamous cell carcinoma (SCCa) transformation and other head and neck malignancies [9]. A few studies have suggested that inverted papillomas with SCCa may exhibit higher standardised uptake values (SUVs) than those without squamous cell carcinoma (SCCa) [8,10]. According to Krouse JH, 9.1% of patients with inverted papillomas get cancer [12].

The primary treatment approach for inverted papilloma (IP) is surgery, which aims to alleviate symptoms and provide a complete specimen for pathological examination to detect the presence of carcinoma. Preoperative medical treatment may involve antibiotics and corticosteroids to reduce inflammation and bleeding during surgery, although the effectiveness of this approach is still uncertain. Successful treatment relies on completely exposing attachment of tumor site to ensure total resection. Recurrences of IP often occur within 2 years after surgery, typically at the original lesion site [1].

A detailed preoperative examination of the extent and location of origin of the inverted papilloma (IP) is critical for planning surgery and evaluating prognosis. Different stagings have been created throughout the years to guide the evaluation of IP, particularly with the improvement of endoscopic procedures. Given that it links the prognosis of IP with the complexity of the surgical treatment, Krouse's staging system is frequently utilised for radiographic staging [13]. This method does have some drawbacks, though, as it classifies T4 lesions as inverted papilloma with extranasal or extrasinus extension and those with a squamous cell carcinoma (SCCa) emphasis. Prognosis and surgical management between these two groups of IP can vary greatly. Based on recurrence rates, a more modern three-group staging approach developed by Cannady et al. offers more prognostic data [14]. With a low recurrence rate of just 3%, Group A contains IP that is restricted to the nasal cavity, ethmoid sinus, and medial maxillary sinus. Group B includes IP that affects the frontal, sphenoid, and lateral maxillary sinuses and has a higher recurrence rate of 19.8%. Group C represents extrasinus-extended IP, which has a 35.3% recurrence rate. However, neither the Cannady system nor the Krouse system independently evaluate the effect of cancer on prognosis [13]. Accurate staging of IP is crucial for selecting the most suitable surgical approach and predicting the likelihood of recurrence. A comprehensive evaluation of radiographic findings and the presence of malignancy should be taken into consideration to make informed treatment decisions and provide accurate prognostic information [8]. In cases where Inverted papilloma is associated with carcinoma or when surgery is not feasible, radiation therapy (RT) may be considered as an alternative treatment option [1]. Ward made the initial discovery of an inverted papilloma in the sinonasal cavity in 1854 [15]. Hyams then further classified sinonasal papillomas into three different cell types: cylindrical, fungiform, and inverted. This rare tumor accounts for between 0.5 and 4% of all primary nasal tumors, with 0.6 cases per 100,000 people annually [4,16,17].

## Conclusions

Inverted sinonasal papillomas are uncommon benign tumors primarily originating from the Schneiderian membrane. While they typically develop from the lateral walls of the nasal cavity and the paranasal sinuses, they are uncommon to develop from the nasal septum. This case report highlights the unusual occurrence of an inverted sinonasal papilloma from the nasal septum, involving the anterior cartilaginous part and resulting in mucosal defects and cartilage destruction. Awareness of this uncommon presentation and its potential implications is critical for accurate diagnosis, appropriate management, and long-term follow-up of patients with sinonasal papillomas. Cases of inverted papillomas originating from nasal septum are fair and far between rare.
